# “Childhood Anemia in India: an application of a Bayesian geo-additive model”

**DOI:** 10.1186/s12887-021-03008-0

**Published:** 2021-11-30

**Authors:** Holendro Singh Chungkham, Strong P. Marbaniang, Pralip Kumar Narzary

**Affiliations:** 1 Indian Statistical Institute, North-East Centre, Tezpur, Assam India; 2 Stress Research Institute, Stockholm University, Stockholm, Sweden; 3 Department of Public Health & Mortality studies, International Institute for Population Sciences, Mumbai, Maharashtra India; 4 Department of Statistics, Sankardev College, Shillong, Meghalaya India; 5 Department of Geography, Bodoland University, Kokrajhar, Assam India

**Keywords:** Spatial effects, Geo-additive logistic regression, P-splines, Childhood anaemia

## Abstract

**Background:**

The geographical differences that cause anaemia can be partially explained by the variability in environmental factors, particularly nutrition and infections. The studies failed to explain the non-linear effect of the continuous covariates on childhood anaemia. The present paper aims to investigate the risk factors of childhood anaemia in India with focus on geographical spatial effect.

**Methods:**

Geo-additive logistic regression models were fitted to the data to understand fixed as well as spatial effects of childhood anaemia. Logistic regression was fitted for the categorical variable with outcomes (anaemia (Hb < 11) and no anaemia (Hb ≥ 11)). Continuous covariates were modelled by the penalized spline and spatial effects were smoothed by the two-dimensional spline.

**Results:**

At 95% posterior credible interval, the influence of unobserved factors on childhood anaemia is very strong in the Northern and Central part of India. However, most of the states in North Eastern part of India showed negative spatial effects. A U-shape non-linear relationship was observed between childhood anaemia and mother’s age. This indicates that mothers of young and old ages are more likely to have anaemic children; in particular mothers aged 15 years to about 25 years. Then the risk of childhood anaemia starts declining after the age of 25 years and it continues till the age of around 37 years, thereafter again starts increasing. Further, the non-linear effects of duration of breastfeeding on childhood anaemia show that the risk of childhood anaemia decreases till 29 months thereafter increases.

**Conclusion:**

Strong evidence of residual spatial effect to childhood anaemia in India is observed. Government child health programme should gear up in treating childhood anaemia by focusing on known measurable factors such as mother’s education, mother’s anaemia status, family wealth status, child health (fever), stunting, underweight, and wasting which have been found to be significant in this study. Attention should also be given to effects of unknown or unmeasured factors to childhood anaemia at the community level. Special attention to unmeasurable factors should be focused in the states of central and northern India which have shown significant positive spatial effects.

## Background

Anemia among children is still a major public health concern in both developed and developing countries. Anemia is a condition in which the number and size of red blood cells or haemoglobin concentration is lower than the established cut-off value [1]. Haemoglobin is essential to carry oxygen and if the body has abnormal or low red blood cells or not enough haemoglobin level, there will be a reduced capacity of the blood to carry oxygen to the body tissues. Globally, anemia affects 1.6 billion people, of which 47.4% were preschool-age children [2]. According to the World Health Organization, (2008), anemia is considered a severe public health problem if the prevalence is 40% or more [2]. In India, 58.5% percent of children between the age of 6 months to 5 years were anemic during 2015–2016 [3]. Moreover, studies have acknowledged high prevalence of anaemia in low and middle-income countries [4], with 67.6 and 65.6% preschool-age children in Africa and South-East Asia suffered from anaemia [2] respectively.

Iron is an essential element of haemoglobin, and iron deficiency is the most common cause of anaemia. However, deficiency in micronutrient-rich diet, Vitamin A, and Vitamin B12 could be the reason for iron deficiency [5]. Also, diseases like diarrhea [6], malaria [7], helminth infection, and hookworms [5] increases the risk of anemia. In India, due to various socio-economic, cultural, and religious beliefs, dietary food habits also vary across the population. Dietary pattern is an essential factor associated with iron intake and absorption. For example, a vegetarian diet may increase the risk of anemia due to lack of iron fortification [8]. Existing literature have also shown that socio-economic factors such as lower maternal education, low economic status [9], and demographic factors such as age and sex of a child [10] affect anaemia. Maternal health status during pregnancy had a significant impact on the health and nutritional status of the child. Evidence from previous studies reported that maternal anaemia, and child nutritional statuses such as wasting, stunting and underweight increase the risk of anaemia [11, 12]. During the first 5 years of life, children are most vulnerable to iron-deficiency anaemia because of increased iron requirements due to their rapid growth [13]. Iron deficiency in children is a serious concern because it may increase childhood morbidity, impaired growth development, and have long term effects on cognitive development and school performance [13].

Accounting for geographical heterogeneity of anaemia and the possible cause of heterogeneity is vital for the allocation of health resources to prevent and control anaemia. Geographical heterogeneity can be an effect of an unobserved independent variables which may include contextual factors. According to Koissi & Högnäs, (2013) ignorance of geographical heterogeneity due to unobserved characteristics could lead to biased estimation of parameters [14]. Geographical heterogeneity could be the effect of the unmeasured factors, which means that the geographical differences of factors that caused anaemia can be partially explained by the variability in environmental factors [15]. Environmental factors such as availability of toilet facility, type of house, source of drinking water, seasonality influence the risk of anaemia among children. Studies found that lower odds of anemia among children living in household with better toilet facility, improve drinking water and better housing condition [16]. Malaria which causes anaemia is known to be associated with altitude and weather conditions such as temperature and rainfall [17]. Similarly, soil-transmitted helminth infection, which causes anaemia is influenced by the distance to water bodies, surface temperature, index of vegetation and rainfall [18]. There are number of studies using different statistical models such as multilevel and spatial mixed model to determine the effect of geographical heterogeneity on childhood anaemia in India [9, 10]; however, all these studies have overlooked the advantage of using bivariate spline in modelling geographical heterogeneity. Above models failed to explain especially the non-linear effect of continuous covariates on childhood anaemia. Thus, the pioneering contribution of this study would be to explore correlated spatial effect of anaemia among children aged 6 to 59 months using the spatial mixed model by assuming the flexible approach of bivariate splines. This study would probably be the first in India to map childhood anaemia in terms of residual spatial effects due to unmeasured factors. So, the map would have important implications for targeted policy for allocation of resources and to search for unmeasured variables that are responsible for residual spatial effects.

## Methods

### Study area

The study used the fourth round of the Indian National Family and Health Survey (2015–2016) which adopted a multi-stage stratified cluster sampling design [19]. From all over India, total of 699,686 eligible women between 15 and 49 years of age completed the interview. The data for the present study uses child as the unit of analysis, rather than the mother. Information was available on 259,627 children born in the last 5 years preceding the survey. The present study excluded the two union territories i.e., Andaman & Nicobar Island and Lakswdeep as their borders are not connected to other parts of India and which would create problem in the estimation of spatial effects. Children with missing haemoglobin level were also dropped from the analysis. With this criterion the final analytical sample size consists of children 208,707.

### Outcome variable and covariates

The outcome variable used in the analysis was based on the categorization of haemoglobin level of children adjusted for altitude. The children whose haemoglobin level was less than 11Hb categorised as being anaemic otherwise not anaemic. The covariates in the present study were selected based on previous study [15] and theoretical understanding of the issue under investigation. As such, mother’s educational level, anaemia status, age and duration of breast feeding are considered as co-variates. Children related characteristics considered are whether children had cough, had fever, received vitamin A, whether stunting, wasting, underweight, birth weight, birth order, and age of the children. Further, household wealth index and family size are included in the study. Duration of breast feeding, age of children, and mother’s age were treated as continuous variables. However, the standard -2SD cut off values of z-scores categorization of height for age, weight for height, and weight for age were used to characterize stunting, wasting and underweight respectively.

### Statistical analysis

Multiple logistic regression model was employed to select potential covariates for childhood anaemia prior to spatial analysis. A significance level of 20% was set for the selection of potential covariates to allow for selection of more variables to be used in the further analysis of spatial modelling. Geo-additive logistic regression models were fitted to the data to understand fixed as well as spatial effects of childhood anaemia. Basically, the model takes the form of a multiple variable hierarchical model as

$$g\left(\mu \right)= U\beta +{e}_i$$

Where *g* is the logit link function which gives the log odds of being anaemic and it links the mean of the response to the predictor *Uβ* + *e*_*i*_, and *e*_*i*_ is the area level random effects representing unmeasured contextual factors. More formally, we can formulate the above model as, if *p*_*ij*_ is the probability that child *j* from location *i* being anaemic, then child anaemic status which is binary is distributed as *Bernoulli*(*p*_*ij*_). Then, following models were fitted to estimate fixed and spatial effects.

$$M0: logit\left({p}_{ij}\right)={z}_i^{\prime}\beta$$

$$M1: logit\left({p}_{ij}\right)={z}_i^{\prime}\beta +{f}_1\left({u}_{i1}\right)+{f}_1\left({u}_{i2}\right)+\dots +{f}_1\left({u}_{ip}\right)$$

$$M2: logit\left({p}_{ij}\right)={z}_i^{\prime}\beta +{f}_{spatial}\left({S}_i\right)$$

$$M3: logit\left({p}_{ij}\right)={z}_i^{\prime}\beta +{f}_1\left({u}_{i1}\right)+{f}_1\left({u}_{i2}\right)+\dots +{f}_1\left({u}_{ip}\right)+{f}_{spatial}\left({S}_i\right)$$

All categorical and continuous variables were treated as fixed effects in *M0*. In case of *M1*, categorical variables were employed as fixed effects and continuous variables were modelled by non-parametric smooth functions *f*_*j*_*s*. Model *M2* included a spatial effect of the state where a child belongs in addition to the fixed effects of categorical variables. Finally, *M3* was a combination of *M1* and *M2*. The smooth functions *f*_*j*_*s* were specified as Bayesian splines and can be approximated by polynomial spline priors of degree *l* at equally spaced knots ${u}_j^{min}={\gamma}_{j0},{\gamma}_{j1},\dots \dots, {\gamma}_{js}={u}_j^{max}$ which are within the domain of covariate *u*_*j*_, and the spatial component *f*_*spatial*_(*S*_*i*_) with Markov random field prior [20, 21] which captures the area of the child random effect. The Bayesian spline can be expressed as a linear combination of *d = s + l* basis functions *B*_*m*_ having the form as,

$${f}_j\left({u}_j\right)=\sum_{m=1}^d{\varepsilon}_{jm}{B}_m\left({u}_j\right)$$

Then, the Bayesian estimation of the above spline reduces to estimating model parameters *ε*_*j*_*s* by assigning first or second order random walk priors for the regression coefficients. A tensor product of two-dimensional spline has been used to model the spatial effect as,

$${f}_{spatial}\left({u}_1,{u}_2\right)=\sum_i^k\sum_j^k{B}_{spatial, ij}{B}_{1i}\left({u}_1\right){B}_{2j}\left({u}_2\right)$$

where, the combination (*u*_1_, *u*_2_) corresponds to the coordinates of the location of the data point, latitude and longitude, or the location centroids based on the map. The commonly available spatial smoothness priors in spatial statistics [22] based on the four nearest neighbours have been adopted.

A fully integrated Bayesian approach was adopted to estimate the parameters and the estimated posterior odds ratio (OR) can be interpreted as the odds ratio from the logistic regression models. The models were fitted using the freely available package *bamlss* [23] in *R* (*R* Core Team, 2020). A total of 40,000 MCMC iterations and 10,000 number of burn in samples were used in the analysis. Convergence of models were checked through autocorrelations and sampling paths. Finally, models were compared by Deviance Information Criterion (*DIC*) values [24], where the model with the smallest value is the preferred one. The *DIC* is calculated as $DIC=\overline{D}+{p}_D$, where $\overline{D}$ is the posterior mean of the model deviance, which gives a measure of goodness of fit, and *p*_*D*_ is the effective number of parameters describing the complexity of the model and controls for penalty for model overfitting.

## Results

### Descriptive results

Table 1 provides prevalence of childhood anaemia according to region and states in India. Northern, central, and eastern regions show high prevalence of anaemia compared to other regions. The prevalence is above 60% in these three regions. The states of Chandigarh and Haryana show relatively high prevalence of anaemia of about 73 and 72% in northern region. In the central region, Madhya Pradesh and Uttar Pradesh show relatively high prevalence of anaemia. Jharkhand and Bihar are the states in eastern region having relatively high prevalence of anaemia of about 70 and 64% respectively. Most of the states in the north-eastern region show comparatively low prevalence of anaemia ranging from 24 to 57%. The states of Karnataka and Telangana show relatively high prevalence of anaemia above 60%. The overall prevalence of anaemia in India is about 58%.

**Table 1:** State variation of childhood anaemia

Region/State	Percentage of children (Anaemic)	Number of cases with anaemic children
**Northern**	62.2	21,765
Chandigarh	72.7	112
Haryana	72.3	4725
Himachal Pradesh	58.1	1324
Jammu and Kashmir	59.6	3986
Delhi	61.3	627
Punjab	57.3	2544
Rajasthan	60.9	8447
**Central**	63.0	41,351
Chhattisgarh	42.9	3060
Madhya Pradesh	69.7	14,015
Uttar Pradesh	63.8	21,468
Uttarakhand	59.1	2808
**Eastern**	61.2	27,158
Bihar	63.6	13,332
Jharkhand	70.1	7002
Odisha	48.6	4393
West Bengal	55.6	2431
**North-Eastern**	35.8	10,504
Arunachal Pradesh	53.3	1956
Assam	35.7	2838
Manipur	24.2	1153
Meghalaya	48.7	1706
Mizoram	23.9	975
Nagaland	26.2	908
Sikkim	56.6	457
Tripura	48.1	511
**Southern**	54.7	10,806
Andhra Pradesh	58.2	1246
Karnataka	62.1	3818
Kerala	36.0	742
Puducherry	43.8	408
Tamil Nadu	51.5	3461
Telangana	64.3	1131
**Western**	58.2	8524
Dadra & Nagar Haveli	83.9	220
Daman & Diu	72.4	205
Goa	48.3	174
Gujarat	63.7	3839
Maharashtra	52.9	4086
**India**	57.6	120,108

Table 2 provides a comparison of childhood anaemia across categorical covariates and a test of significance difference between categories of each covariate by chi-square test. It is evident that children from rural, mother with low education, household of poor economic condition show higher prevalence of anaemia than their respective counterparts. There is a clear significant difference in childhood anaemia by place of residence, mother’s education and household wealth. But no significant difference in childhood anaemia by sex of child is observed. Children with fever show a tendency of higher prevalence of anaemia. It can also be seen that consumption of vitamin A supplement during childhood is helpful to reduce prevalence of anaemia. Under nutrition of children also show an increase in prevalence of anaemia. At 5% level of significance the categorical variables- place of residence, mother’s education, mother’s anaemic status, household economic status, children’s fever, vitamin A, stunting, wasting, and underweight are associated with childhood anaemia without controlling for other covariates. The categorical variables children’s birth order, children’s birth weight and household size show a non-significant effect on childhood anaemia at 20% level of significance in the preliminary analysis. Therefore, only categorical variables listed in Table 2 are included in the spatial logistic regression model in Table 4.

**Table 2:** Prevalence of childhood anaemia by fixed covariates with effect coding used in model

Factor	N (%)	P*	Effect coding
Place of residence		< 0.001	
Urban	27,338 (55.2)		1
Rural	92,770 (58.3)		-1^R^
Sex of the child		0.644	
Male	62,486 (57.5)		1
Female	57,622 (57.6)		-1^R^
Mother’s education		< 0.001	
Primary	17,845 (58.3)		1
Secondary	50,460 (54.1)		2
Higher	9467 (50.1)		3
No education	42,336 (64.3)		-1^R^
Wealth index		< 0.001	
Poor	28,395 (57.6)		1
Middle	23,422 (56.2)		2
Rich	18,677 (53.9)		3
Richest	14,804 (52.9)		4
Poorest	34,810 (63.2)		-1^R^
Fever		< 0.001	
Yes	16,729 (60.9)		1
No	103,295 (57.1)		-1^R^
Missing	84 (52.8)		
Cough		0.220	
Yes	13,887 (57.1)		1
No	106,159 (57.6)		-1^R^
Missing	62 (54.9)		
Child received vitamin A		> 0.001	
Yes	38,674 (58.1)		1
No	80,003 (57.3)		-1^R^
Missing	1431 (57.1)		
Stunting		< 0.001	
Yes	50,438 (62.7)		1
No	64,015 (53.6)		-1^R^
Missing	5655 (63.9)		
Underweight		< 0.001	
Yes	45,252 (63.7)		1
No	69,201 (53.7)		-1^R^
Missing	5655 (63.9)		
Wasting		< 0.001	
Yes	8814 (64.1)		1
No	105,639 (56.8)		-1^R^
Missing	5655 (63.9)		
Mother anaemic		< 0.001	
Yes	48,928 (67.8)		1
No	70,787 (52.1)		-1^R^
Missing	393 (58.1)		

^R^: Reference category; *: *p*-value of chi-square test of independence

### Model selection

The selection of the most preferred model is based on the deviance information criterion (DIC) and deviance values. Model with the smallest values of DIC and deviance is the preferred model. With these criteria, model *M3* is the preferred model (Table 3). Therefore, interpretations of results (Table 4) and discussions are based on model *M3*.

**Table 3:** Model comparison by deviance information criterion (DIC)

Model Fit	Deviance	pD	DIC
M0	171,173.90	19.79	171,154.10
M1	170,885.30	37.71	170,847.60
M2	165,233.90	51.77	165,182.10
M3	164,909.50	69.92	164,839.60

**Table 4:** Fixed effects on childhood anaemia in India

Variable	Mean	SD	10%	Median	90%
Place of residence					
Rural^R^					
Urban	0.0359*	0.008	0.0262	0.0355	0.0461
Sex of child					
Female^R^					
Male	0.0074	0.006	−0.0003	0.0075	0.0148
Mother’s education					
No education^R^					
Primary	0.0563*	0.014	0.0386	0.0564	0.0740
Secondary	−0.0358*	0.010	−0.0481	− 0.0361	− 0.0229
Higher	− 0.1843*	0.016	− 0.2056	− 0.1844	− 0.1625
Wealth index					
Poorest^R^					
Poor	0.0740*	0.012	0.0585	0.0736	0.0893
Middle	0.0069	0.012	− 0.0079	0.0071	0.0222
Rich	−0.0904*	0.013	−0.1072	− 0.0904	− 0.0736
Richest	− 0.1332*	0.017	−0.1548	− 0.1330	− 0.1125
Child had fever					
No^R^					
Yes	0.0326*	0.010	0.0200	0.0327	0.0451
Child had cough					
No^R^					
Yes	−0.0594*	0.010	−0.0723	− 0.0596	− 0.0466
Child received vitamin A					
No^R^					
Yes	−0.0041	0.007	−0.0125	− 0.0042	0.0042
Child stunted					
No^R^					
Yes	0.0999*	0.007	0.0903	0.1000	0.1091
Child underweight					
No^R^					
Yes	0.0797*	0.008	0.0698	0.0795	0.0899
Child wasted					
No^R^					
Yes	0.0387*	0.012	0.0235	0.0387	0.0541
Mother anaemic					
No^R^					
Yes	0.2715*	0.007	0.2632	0.2714	0.2803

^R^: Reference category. *:Statistically significant at 5% alpha

### Fixed effects

Table 4 shows fixed effects to childhood anaemia. Place of residence, mother’s education, poorest, rich, richest categories of household wealth, fever, cough, child under nutrition and mother’s anaemic status are fixed effects variables which are significant to childhood anaemia. The fixed effects coefficient for fever is positive, which indicates that children with fever are likely to increase the risk of childhood anaemia. Children who take vitamin A supplement decrease the likelihood of becoming anaemic. Children from rich or richest quintile of household wealth also have lesser risk of childhood anaemia than those who belong to poorest quintile. Children who are malnourished increase the risk of childhood anaemia. Mother’s anaemic status has a positive effect on childhood anaemia. This means that children whose mothers are anaemic have higher risk of being anaemic than those whose mothers are not anaemic.

### Non-linear effects

Another reason behind the geo-additive modelling is the ability to incorporate non-linear effects of continuous variables in the model. In the present study, we incorporated non-linear effects of age of child, mother’s age and, duration of breast feeding.

**Fig. 1:**
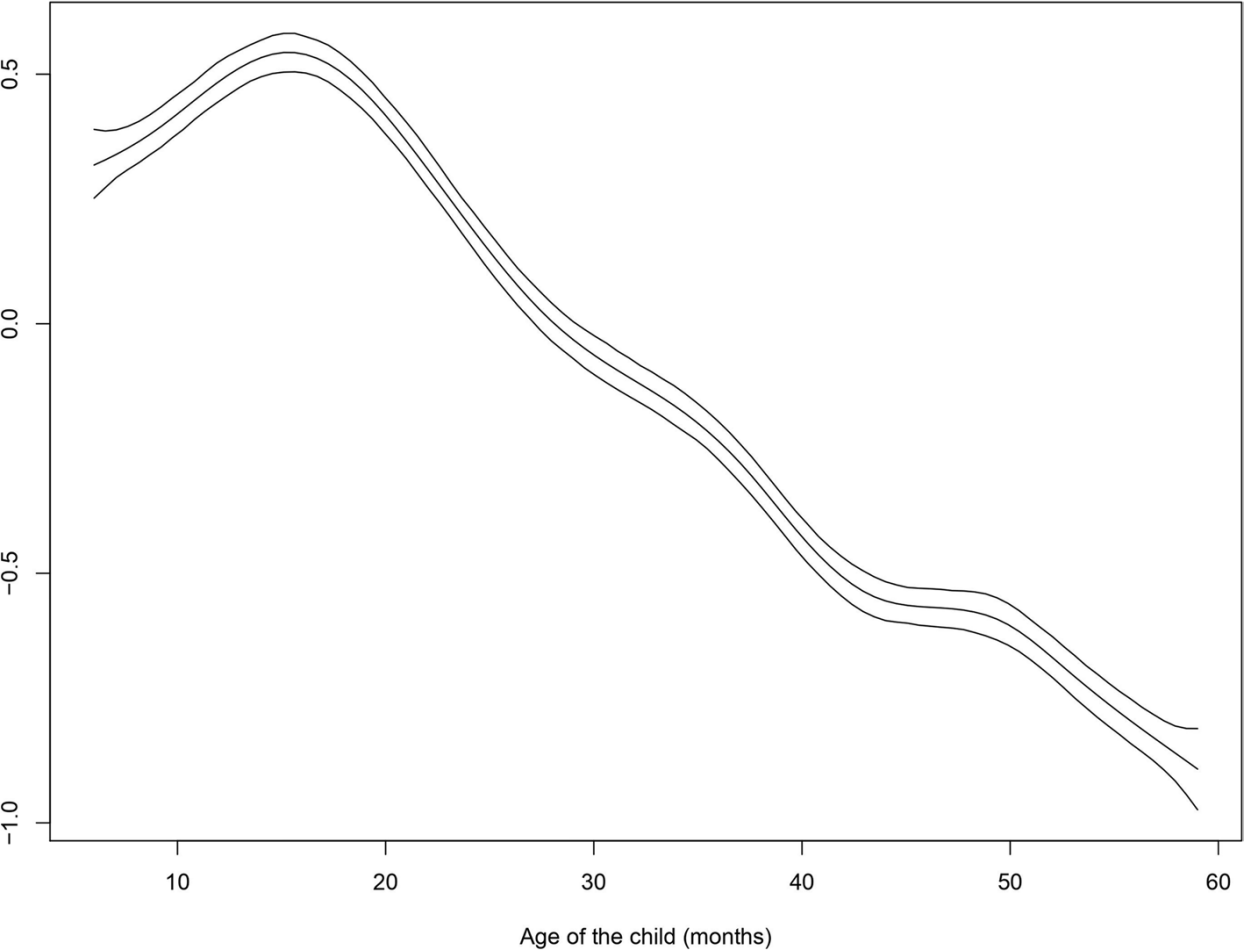
Non linear effect of age of the children on childhood anaemia. Lower and Upper lines indicate 95% confidence interval

The age of children has non-linear effect on childhood anaemia (Fig. 1). It is evident from Fig. 1 that as the age of children increases, its effect on childhood anaemia decreases, which indicates, older children are less likely to have the risk of childhood anaemia. The risk of having anaemia is much higher among younger children aged about 6 months to about 15 months and decreases thereafter.

**Fig. 2:**
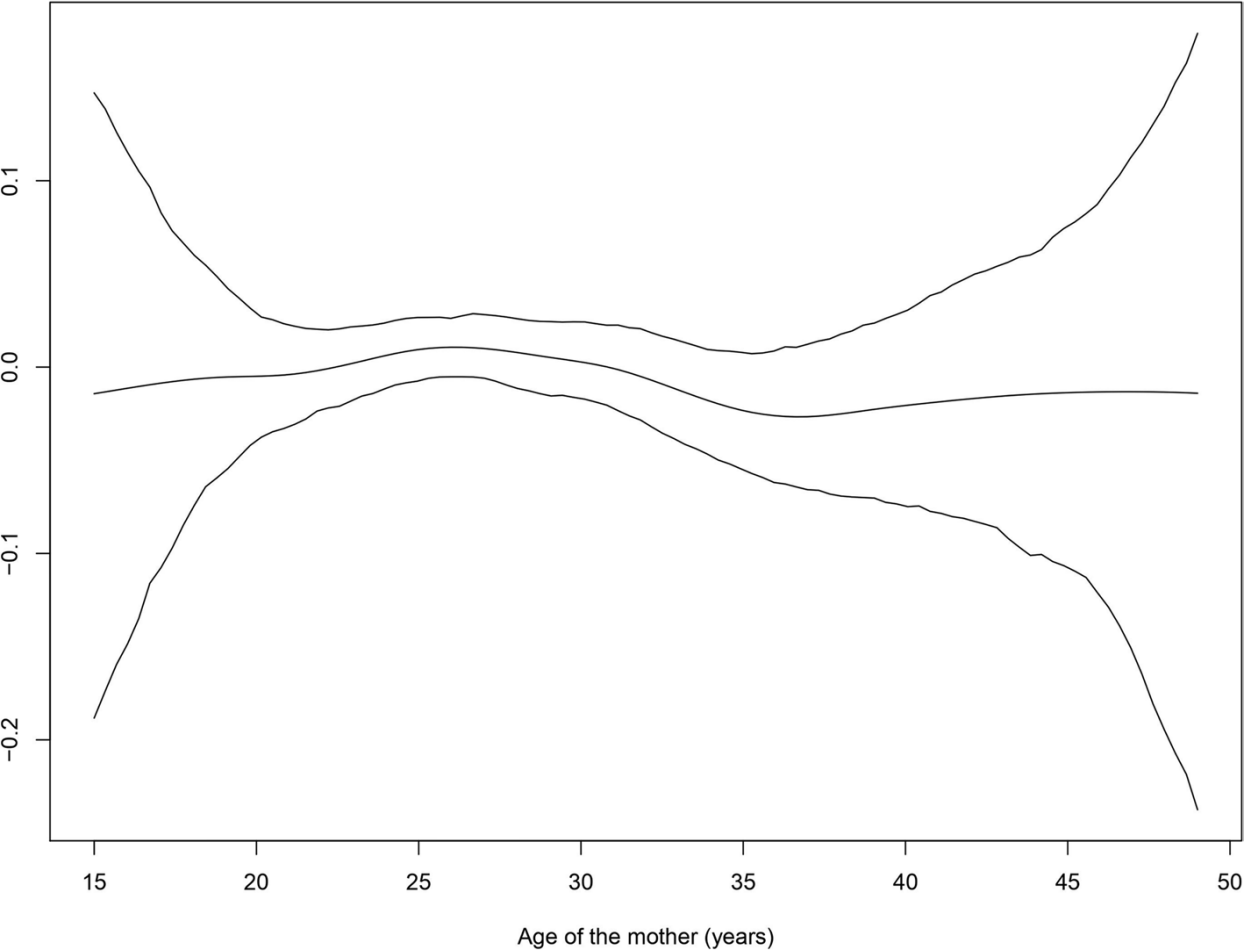
Non linear effect of mother’s age on childhood anaemia. Lower and Upper lines indicate 95% confidence interval

Mother’s age also has a non-linear effect on childhood anaemia (Fig. 2). The functional relationship between childhood anaemia and mother’s age depicts almost a U shape pattern. This indicates that mothers of young (in particular mothers aged 15 years to about 25 years) and old ages are more likely to have children who are anaemic. The risk of childhood anaemia starts declining after the age of 25 years and continuous till the age of around 37 years, thereafter again starts increasing.

**Fig. 3:**
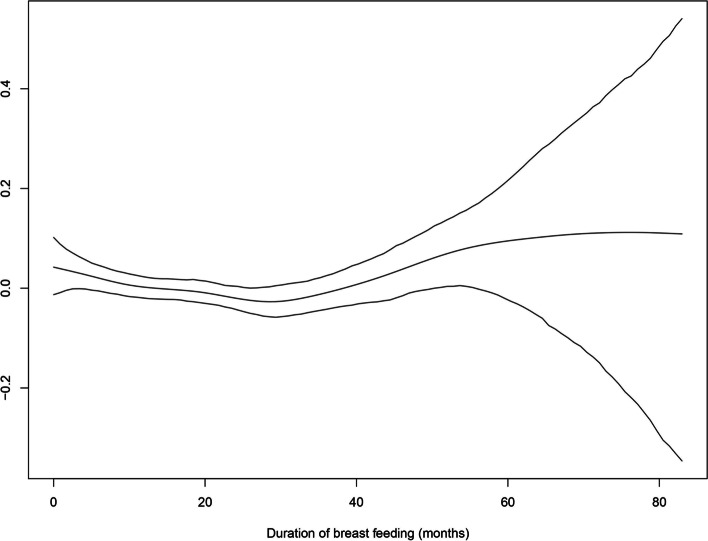
Non linear effect of duration of breast feeding on childhood anaemia. Lower and Upper lines indicate 95% confidence interval

Figure 3 shows the non-linear effects of duration of breast feeding on childhood anaemia. The risk of childhood anaemia decreases till 29 months, thereafter increases. This indicates improvement in childhood anaemia with increase in duration of breast feeding. The credible intervals are wider at extreme ages because of small cases of observations.

### Spatial effects

**Fig. 4:**
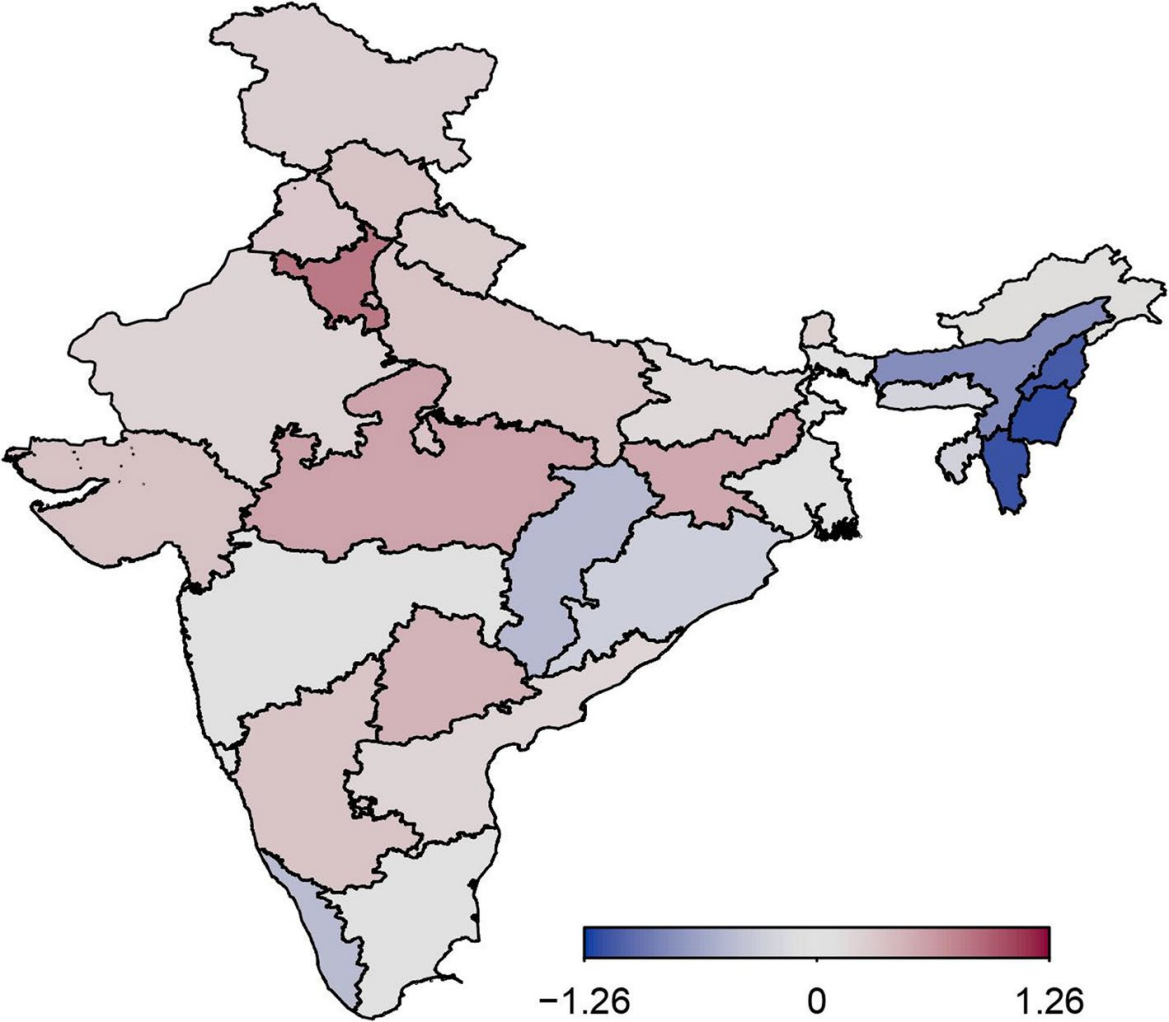
Residual spatial effect to childhood anaemia. Colour ranges from blue to red representing low to high risk of childhood anaemia. Source of Shapefile Map: Bhuvan India Geo Platform of Indian Space Research Organisation, Govt. of India

**Fig. 5:**
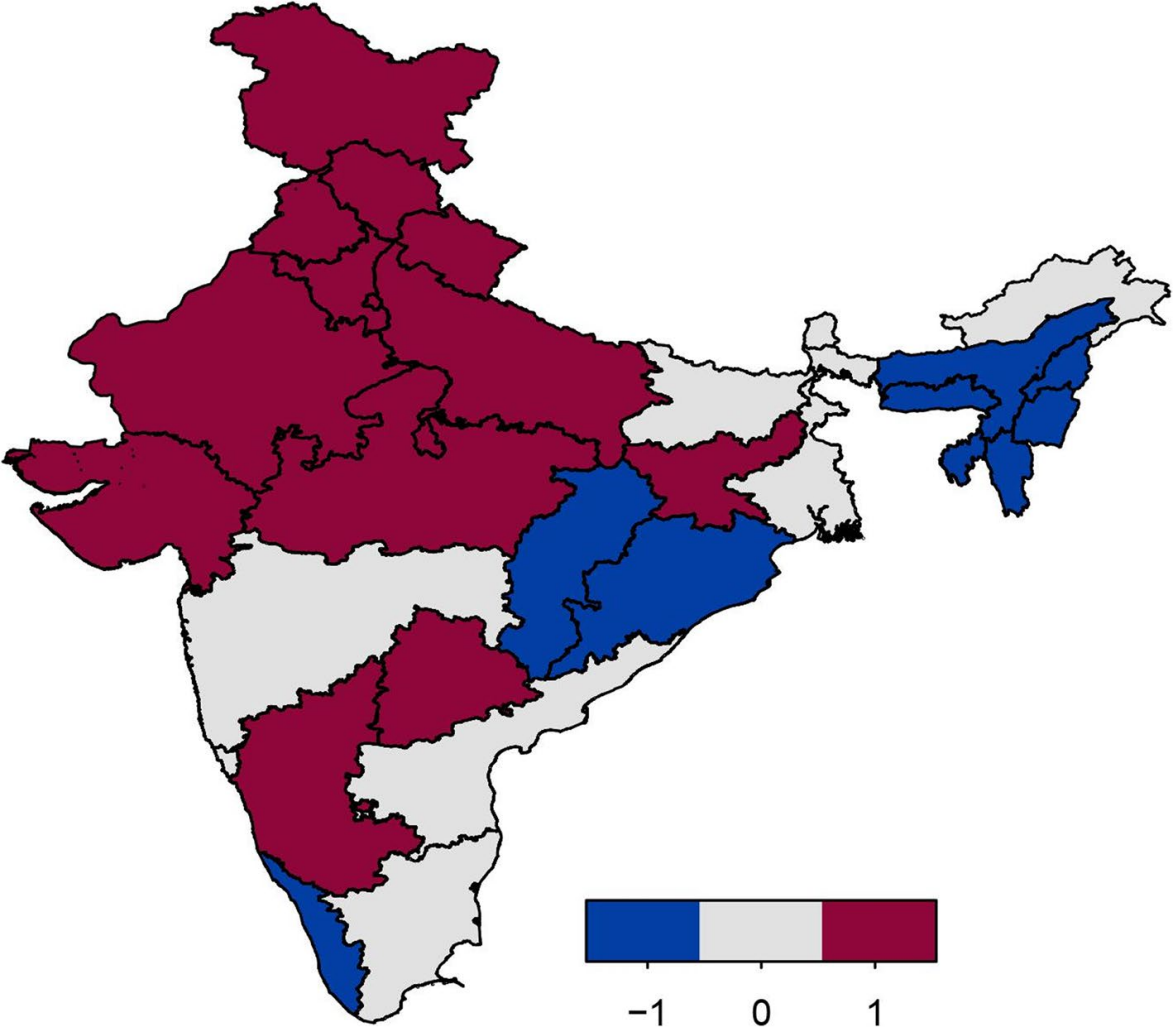
The 95% credible intervals map for prevalence of anaemia. Blue: negative effect; light gray: insignificant effect; red: positive effect. Source of Shapefile Map: Bhuvan India Geo Platform of Indian Space Research Organisation, Govt. of India

**Fig. 6:**
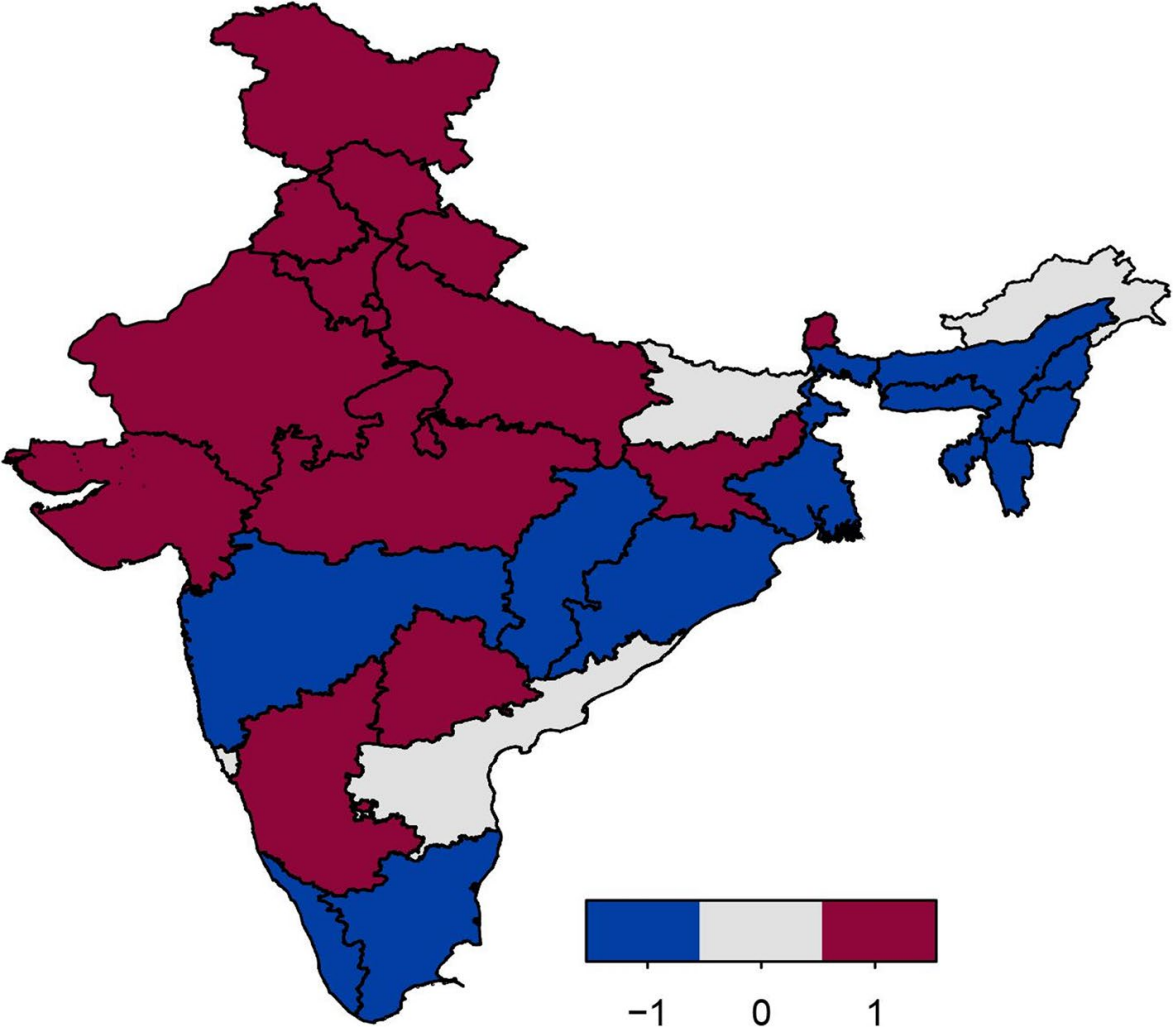
The 80% credible intervals map for prevalence of anaemia. Blue: negative effect; light gray: insignificant effect; red: positive effect. Source of Shapefile Map: Bhuvan India Geo Platform of Indian Space Research Organisation, Govt. of India

Figure 4 displays the estimates of the spatial effects of childhood anaemia, with colour range goes from blue to red representing low to high risk of childhood anaemia. Spatial effects represent unobserved influences, such as environmental and climatic factors, availability of good transport facility, and access to good services for child health. The figure clearly shows evidence of residual spatial effects of childhood anaemia in India with most of states showing significant positive/negative effects with respect to the 95% posterior credible interval map (Fig. 5). With respect to 80% posterior credible interval more states show significant spatial effects (Fig. 6). Most of the states in northern and central regions show significant positive spatial effects with respect to 95% credible interval. However, almost all states in north-eastern region of India show significant negative spatial effects with regard to the 80% credible interval (Fig. 6).

## Discussion

In India Childhood anaemia cuts across all the sections of society with varying intensity. Its prevalence, as per the WHO classification, is a severe public health problem for India. Except for Mizoram, Manipur, Nagaland, Assam, and Kerala for all the states and union territories (UTs,) anaemia is a matter of concern, whereas for states like Haryana, Jharkhand, and Madhya Pradesh it is of extremely serious concern. These three states need to revisit existing programs targeting to address the child health in general and anaemia in particular.

Anaemia has a close link with the food habit. Food habit is closely associated with culture and the nature. Geographical settings decide the nature of food supply and the micronutrients. Within the same geographical settings culture may encourage or discourage some group of population to consume or avoid certain nutritious food. For example tribal culture of northeast India approves consumption of varieties of insects, whereas for non-tribals consumption of such insects is considered as taboo. Probably because of this reason the tribal dominated states like Mizoram, Manipur, and Nagaland have very low prevalence of anaemic children. However, our finding contradicts other studies in India that children from lowest socioeconomic strata have more likelihood of suffering from anaemia [9, 25] and Nepal [26].

The prevalence of anaemia among children in rural areas is comparatively higher than their counter part in India. Rural mass in India might be less aware about the balanced diet which has potentials to improve the hemoglobin count. Because, as high as one third of rural population in India are illiterate. Ignorance of food items relating to iron content food staff may also add to the problem of anaemia in rural areas. This indicates that mass media campaign to address anaemia should emphasize on pictorial depiction and or audio-visual means, rather than on the written leaflets. A distinct negative relationship between wealth quintile and child anaemia is quite evident. This is indicative of the fact that economically poorer households may not be able to afford to procure food regularly and especially the nutritious food times. This calls for better Public Distribution System (PDS) which provides subsidized food in India. The system needs to keep an eye on mainly on regularity, quantity, and quality of supplies.

Uneducated mothers are less equipped with knowledge of hygiene and proper knowledge of child care. Unhealthy feeding habit can lead to various types of food related health problems. Feeding practice is closely associated with diarrhoeal disease and studies exhibit that there is positive relationship between diarrhoea and anemia. Unlike earlier studies [8, 10] no significant association is noted between sex of the child and prevalence of anaemia in the present study. Children who take vitamin A supplement decrease the likelihood of becoming anaemic. But earlier study [8] did not find significant statistical association between vitamin A intake and childhood anaemia. In India, poor and illiterate families leave their baby on the mud floor. The crawling baby in absence of a care taker may put to mouth anything it comes to her/his hand. Such activities may lead to various infections and morbidities, for which younger children have more likelihood of suffering from anaemia. Other studies also indicate that younger children have more chances of having anaemia [15, 26]. Very young mothers definitely are less educated and relatively old mothers might take child rearing for granted, as they may already have older children and experienced of child rearing. Other study also indicates U-shape relationship between mother’s age and the childhood anaemia [15] and others [10, 27] found children born to young mothers are more likely to be anaemic. In India usually the educated and rich women, due to various reasons, do not practice exclusive breast feeding. Exclusive breast feeding in India is usually practiced among the less educated and poor women, as a result a positive association between exclusive breast feeding and childhood anaemia is observed. However, this finding contradicts studies conducted elsewhere [28].

### Limitations

The present study is not without any limitation despite using an innovative statistical technique. First, our study is based on cross-sectional design. Therefore, control of major confounders and no causal inferences can be made in spite of robustness in the analysis. Second, the study uses only relevant variables in our data set leading to omission of certain important variables such as clamping of umbilical cord after birth mentioned in some studies.

## Conclusions

There is strong evidence of residual spatial effect to childhood anaemia in India. Government child health programme should gear up in treating childhood anaemia by focusing on known measurable factors such as mother’s education, mother’s anaemia status, family wealth status, child fever, stunting, underweight, and wasting which have been found to be significant in this study. Attention should also be given to effects of unknown or unmeasured factors of childhood anaemia at the community level. Special attention to these unmeasurable factors should be focused in the states of central and northern India which have shown significant positive spatial effects. As the problem of anemia is multi-faceted, the Anemia Mukt Bharat strategy adopted under Poshan Abhiyaan shows great hope in bringing down the prevalence of anemia in India by adopting 6x6x6 strategy [29]. The strategy of targeting six groups of population, six interventions, and six institutional mechanisms is very fascinating but only time will tell its success.

## Data Availability

The datasets generated and/or analysed during the current study are available in the Website of Demographic Health Survey https://dhsprogram.com/methodology/survey/survey-display-355.cfm. We submitted a request to the DHS by mentioning the objectives of this study and thereafter was granted the permission to download the dataset.

## Abbreviation

DIC: Deviance information criterion
WHO: World health organisation
OR: Odds ratio
UTs: Union territories
HSC: Holendro singh chungkham
SPM: Strong P marbaniang
PKN: Pralip kumar narzary

## References

[1] Kotwal A (2016). Iron deficiency anaemia among children in South East Asia: determinants, importance, prevention and control strategies. Curr Med Res Pract.

[2] Benoist B de, McLean E, Egli I, Cogswell M, editors. Worldwide prevalence of anaemia 1993-2005: WHO global database on anaemia. World Health Organization; 2008 [cited 2020 Aug 10]. Available from: https://www.who.int/vmnis/anaemia/prevalence/en/

[3] International institute for population sciences (IIPS) and ICF. National Family Health Survey (NFHS-4), 2015–16: India Mumbai:IIPS; 2017. Available from: http://rchiips.org/nfhs/NFHS-4Reports/India.pdf

[4] McLean E, Cogswell M, Egli I, Wojdyla D, de Benoist B (2009). Worldwide prevalence of anaemia, WHO vitamin and mineral nutrition information system, 1993–2005. Public Health Nutr.

[5] Balarajan Y, Ramakrishnan U, Özaltin E, Shankar AH, Subramanian S (2011). Anaemia in low-income and middle-income countries. Lancet.

[6] Howard CT, de Pee S, Sari M, Bloem MW, Semba RD (2007). Association of diarrhea with anemia among children under age five living in rural areas of Indonesia. J Trop Pediatr.

[7] Calis JCJ, Phiri KS, Faragher EB, Brabin BJ, Bates I, Cuevas LE (2008). Severe Anemia in Malawian children. N Engl J Med.

[8] Ray R. Mother’s autonomy and child anemia: A case study from India. Child Youth Serv Rev. 2020;112(March):104537. Available from: https://doi.org/10.1016/j.childyouth.2019.104537.

[9] Sharma H, Singh SK, Srivastava S. Socio-economic inequality and spatial heterogeneity in anaemia among children in India: Evidence from NFHS-4 (2015–16). Clin Epidemiol Glob Heal. 2020;8(4):1158–1171. Available from: https://doi.org/10.1016/j.cegh.2020.04.009.

[10] Dutta M, Bhise M, Prashad L, Chaurasia H, Debnath P. Prevalence and risk factors of anemia among children 6–59 months in India: A multilevel analysis. Clin Epidemiol Glob Heal. 2020 ;8(3):868–878. Available from: https://doi.org/10.1016/j.cegh.2020.02.015.

[11] Rahman MS, Mushfiquee M, Masud MS, Howlader T. Association between malnutrition and anemia in under-five children and women of reproductive age: Evidence from Bangladesh Demographic and Health Survey 2011. Adu-Afarwuah S, editor. PLoS One. 2019;14(7):e0219170. Available from: https://dx.plos.org/10.1371/journal.pone.0219170 10.1371/journal.pone.0219170 PMC6609031 31269082

[12] Engidaye G, Melku M, Yalew A, Getaneh Z, Asrie F, Enawgaw B. Under nutrition, maternal anemia and household food insecurity are risk factors of anemia among preschool aged children in Menz Gera Midir district, eastern Amhara, Ethiopia: a community based cross-sectional study. BMC Public Health 2019;19(1):968. Available from: https://bmcpublichealth.biomedcentral.com/articles/10.1186/s12889-019-7293-0 10.1186/s12889-019-7293-0 PMC6642588 31324244

[13] WHO (2011). Guideline: Intermittent iron supplementation in preschool and school-age children.

[14] Koissi M-C, Högnäs G (2013). Using WinBUGS to study family frailty in Child mortality, with an application to Child survival in Ivory Coast. African Popul Stud.

[15] Ngwira A, Kazembe LN. Bayesian random effects modelling with application to childhood anaemia in Malawi. BMC Public Health 2015 ;15(1):161. Available from: http://bmcpublichealth.biomedcentral.com/articles/10.1186/s12889-015-1494-y 10.1186/s12889-015-1494-y PMC4358301 25885648

[16] Baranwal A, Baranwal A, Roy N. Association of Household Environment and Prevalence of Anemia among children Under-5 in India. Front Public Heal 2014 20;2. Available from: http://journal.frontiersin.org/article/10.3389/fpubh.2014.00196/abstract. 10.3389/fpubh.2014.00196 PMC4202784 25368862

[17] Marbaniang SP, Ladusingh L. Meteorological conditions and malaria cases—study in the context of Meghalaya. In: Issues on health and healthcare in India, India studies in business and economics. Singapore: Springer; 2018. p. 379–393. Available from: http://link.springer.com/10.1007/978-981-10-6104-2_21

[18] Soares Magalhães RJ, Salamat MS, Leonardo L, Gray DJ, Carabin H, Halton K, et al. Mapping the risk of soil-transmitted helminthic infections in the Philippines. Knopp S, editor. PLoS Negl Trop Dis. 2015 ;9(9):e0003915. Available from: https://dx.plos.org/10.1371/journal.pntd.0003915 10.1371/journal.pntd.0003915 PMC4569387 26368819

[19] DHS. The DHS Program-India: Standard DHS, 2015–16 Dataset,. Demographic Health Survey. 2017. Available from: https://dhsprogram.com/data/dataset/India_Standard-DHS_2015.cfm?flag=1

[20] Fahrmeir L, Kneib T, Lang S (2004). Penalized structured additive regression for space-time data: a Bayesian perspective. Stat Sin.

[21] Lang S, Brezger A. Bayesian P-splines. J Comput Graph Stat 2004;13(1):183–212. Available from: http://www.tandfonline.com/doi/abs/10.1198/1061860043010.

[22] Besag J, Kooperberg C (1995). On conditional and intrinsic autoregression. Biometrika..

[23] Umlauf N, Klein N, Zeileis A. BAMLSS: Bayesian additive models for location, scale, and shape (and beyond). J Comput Graph Stat 2018;27(3):612–627. Available from: https://www.tandfonline.com/doi/full/10.1080/10618600.2017.1407325.

[24] Spiegelhalter DJ, Best NG, Carlin BP, van der Linde A. Bayesian measures of model complexity and fit. J R Stat Soc Ser B (Statistical Methodol) 2002;64(4):583–639. Available from: http://doi.wiley.com/10.1111/1467-9868.00353

[25] Goswmai S, Das KK. Socio-economic and demographic determinants of childhood anemia. J Pediatr 2015;91(5):471–477. Available from: http://dx.doi.org/10.1016/j.jped.2014.09.009 10.1016/j.jped.2014.09.009 26070864

[26] Khanal V, Karkee R, Adhikari M, Gavidia T. Moderate-to-severe anaemia among children aged 6–59 months in Nepal: an analysis from Nepal demographic and health survey, 2011. Clin Epidemiol Glob Heal 2016;4(2):57–62. Available from: http://dx.doi.org/10.1016/j.cegh.2015.07.001

[27] Onyeneho NG, Ozumba BC, Subramanian SV (2019). Determinants of childhood Anemia in India. Sci Rep.

[28] Dalili H, Baghersalimi A, Dalili S, Pakdaman F, Hassanzadeh Rad A, Abbasi Kakroodi M (2015). Is there any relation between duration of breastfeeding and anemia?. Iran J Pediatr Hematol Oncol.

[29] National Health Mission (NHM). Ministry of Health and Family Welfare, Government of India, "6 Interventions of the Anemia Mukt Bharat Programme," 2021. Available from https://anemiamuktbharat.info/home/interventions/. Accessed 21 Sep 2021

